# Fingerprint analysis of Resina Draconis by ultra-performance liquid chromatography

**DOI:** 10.1186/s13065-017-0299-8

**Published:** 2017-07-24

**Authors:** Yudi Xue, Lin Zhu, Tao Yi

**Affiliations:** School of Chinese Medicine, Hong Kong Baptist University, Kowloon Tong, Hong Kong Special Administrative Region People’s Republic of China

**Keywords:** Resina Draconis, UPLC, Chromatographic fingerprint, Similarity

## Abstract

**Background:**

Resina Draconis, a bright red resin derived from *Dracaena cochinchinensis*, is a traditional medicine used in China. To improve its quality control approach, an ultra-performance liquid chromatography (UPLC) fingerprint method was developed for rapidly evaluating the quality of Resina Draconis.

**Methods:**

The precision, repeatability and stability of the proposed UPLC method were validated in the study. Twelve batches of Resina Draconis samples from various sources were analyzed by the present UPLC method. Common peaks in the chromatograms were adopted to calculate their relative retention time and relative peak area. The chromatographic data were processed by Similarity Evaluation System for Chromatographic Fingerprint of Traditional Chinese Medicine software (Version 2004 A) for similarity analysis.

**Results:**

The present UPLC method demonstrated a satisfactory precision, repeatability and stability. The analysis time of the present UPLC method was shortened to 30 min, compared with that of the conventional HPLC method was 50 min. The similarities of the 12 Resina Draconis samples were 0.976, 0.993, 0.955, 0.789, 0.989, 0.995, 0.794, 0.994, 0.847, 0.987, 0.997, 0.986, respectively, which indicated that the samples were certainly regionally different. The similarities of the 12 samples showed more similar pattern except for samples 4, 7 and 9. Such variation in similarity may presumably be attributed to differences in source.

**Conclusions:**

Compared with the conventional HPLC method, the present UPLC method showed several advantages including shorter analysis time, higher resolution and better separation performance. The UPLC fingerprinting established in the present paper provides a valuable reference for the quality control of Resina Draconis.

**Electronic supplementary material:**

The online version of this article (doi:10.1186/s13065-017-0299-8) contains supplementary material, which is available to authorized users.

## Background

Traditional Chinese medicines (TCMs), which have been used for centuries in China for preventing and treating human diseases, have been gaining more and more global popularity and concern owing to its unique theoretical system and superb efficacy [1]. TCM contains various kinds of herbal medicine and each medicine is composed of complex components which will vary according to many factors including soils, climates, and growth stages [2–4]. Since the therapeutic effects will be influenced by the multiple components of TCM, it is urgent to find a type of quality assessment system to identify species and analysis the complex components of TCM. Chromatographic fingerprint, as a main identification method for the comprehensive control of the quality of TCM, becomes the right research objective [5, 6]. Chinese medicine is multi-component, multi-link, and multi-target and quality control also needs to reflect characteristics of TCM. It’s difficult to measure the quality by only a single or a few indexes. TCM fingerprint, based on a systematic research on the chemical composition of TCM, is a kind of comprehensive, quantifiable identification method which is mainly used for the evaluation of the authenticity, superiority and stability of TCM and semi-finished TCM, and conforms to the integrity and fuzziness characteristics of TCM [7].

Recently, chromatographic technologies, such as thin-layer chromatography (TLC), high-performance liquid chromatography (HPLC), gas chromatograph (GC) and capillary electrophoresis (CE) have been widely used in TCM fingerprint identification [5, 8, 9], among which TLC is a traditional method, fast and easy to operate, but with poor resolution. HPLC is the most common fingerprint method with high precision, sensitivity and repeatability. However, HPLC has the disadvantages of long analysis time, low resolution and big solvent consumption. GC is suitable to volatile compounds. CE is often used for the separation and analysis of solubility in water or alcohol soluble ingredient. CE method is well known for its high separation efficiency, fast analysis speed and low cost, however, the retention time is not stable [10, 11]. Therefore, considering the above factors, a method with fast separation and high resolution was expected in the quality control of TCM. Nowadays, UPLC has been gaining popularity in the fast profiling of TCM which is a relatively new technique, and giving new possibilities in liquid chromatography. It managed to save time and solvent consumption [12–16]. As a new type of liquid chromatography, UPLC can significantly improve the degree of separation and detection sensitivity of chromatographic peak, and meanwhile greatly shorten the analysis period, so it is highly suitable for the separation of trace complex mixture and high flux study [15, 16]. At present, UPLC has been applied in many areas such as metabolomics, food safety, illegal addition of drugs, environmental monitoring, quality control of TCM, etc.

Resina Draconis (also called “dragon’s blood”), a bright red resin derived from *Dracaena cochinchinensis*, is a traditional medicine and regarded as a “panacea of blood activation” in China for long [17–19]. It is clinically used to invigorate blood circulation and applicable in the treatment of many diseases including ischemic heart disease, cerebral arterial thrombosis, blood stasis syndrome and traumatic injuries [20]. Resina Draconis is composed of many constituents, of which flavonoids are the main chemical constituents. Besides, stilbenes, saponins, terpenes, phenols and steroids have also been identified as its constituents [19, 21–23]. In the previous studies, the fingerprint of Resina Draconis has been widely analyzed with chromatographic methods and most of the studies are based on HPLC [24, 25]. Nevertheless, the methods were quite time-consuming. Recently, a UPLC method was used to evaluation for the quality of Resina Draconis, however, the analysis time of the method was still up to 45 min [26]. The development of a novel UPLC method remained the primary task for the quality evaluation of Resina Draconis. In this study, a new UPLC method was established for the chromatographic fingerprint validation and quality evaluation of Resina Draconis, aiming to have a better quality control. This experiment investigates the fingerprints of 12 batches of Resina Draconis collected from different regions by UPLC. Meanwhile, the UPLC method is also compared to a HPLC method in order to prove that UPLC method has fast analysis speed, good degree of separation and less required mobile phase, that may provide good reference for the quality control of the dragon’s blood.

## Experimental

### Materials and reagents

Twelve batches of Resina Draconis samples were collected from different regions of China for analysis, and the source information was listed in the Additional file 1: Table S1. The authentication of the samples was identified by Dr. YI Tao according to the morphological features, and the voucher specimens were deposited in the School of Chinese Medicine, Hong Kong Baptist University.

**Fig. 1:**
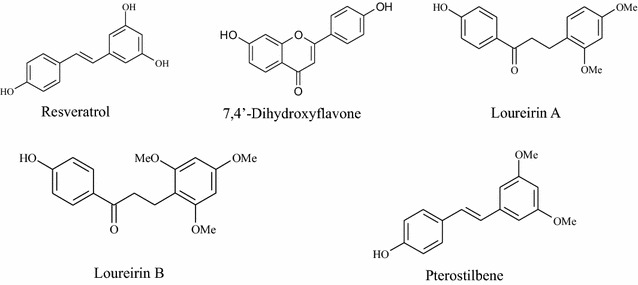
Chemical structures of the reference compounds

Reference compounds of resveratrol, 7,4′-dihydroxyflavone, loureirin A, loureirin B and pterostilbene were provided by the laboratory of quality analysis of TCM, School of Chinese Medicine, Hong Kong Baptist University. The purity of these reference standards was determined to be more than 98% by normalization of the peak areas detected by using a HPLC–DAD system. Their chemical structures were shown in Fig. 1.

Methanol of analytical grade (Labscan, Bangkok, Thailand) was used for preparation of standards and sample solution. Acetonitrile of chromatographic grade (Labscan, Bangkok, Thailand) and deionized water obtained from a Milli-Q water purification system (Millipore, Bedford, MA, USA) were used for preparation of the mobile phase.

### UPLC-PDA instrumentation and conditions

The UPLC system comprised a 500 nL flow cell, an auto sampler, and a photodiode array (PDA) detector. The analysis was carried out by an acquity system from waters and an HSS C_18_ column (2.1 mm × 100 mm, 1.8 μm) was used. For UPLC, the mobile phase was a linear gradient consisting of water (A) and acetonitrile (B) in 30 min. The gradient conditions were: 15–20% (B) at 0–8 min, 20–68% at 8–30 min. The detection wavelength was set at 280 nm and the injection volume was 1.0 μL. The flow rate was 0.3 mL/min, and the column temperature was maintained at 40 °C during the separation.

### HPLC–DAD instrumentation and conditions

The HPLC analysis was carried out by an Agilent 1100 series HPLC–diode array detector (DAD) system comprising a vacuum degasser, binary pump, autosampler, thermostated column compartment, and DAD (Agilent, USA), which was used for acquiring chromatograms and ultraviolet (UV) spectra. An Alltima C_18_ column (4.6 mm × 250 mm, 5 μm) was used for HPLC analysis. The mobile phase consisted of water (A) and acetonitrile (B), and the procedure was performed with a gradient program of 23–27% (B) at 0–18 min, 27– 32% (B) at 18–30 min, 32–33% (B) at 30–35 min and 33–100% (B) at 35–50 min. The flow rate was 1 mL/min. The detection wavelength was set at 280 nm. The column temperature was set at 30 °C. The injection volume of samples and the standard solutions were both 5.0 μL.

### Preparation of the standard solution

Appropriate amount of resveratrol, 7,4′-dihydroxyflavone, loureirin A, loureirin B and pterostilbene were accurately weighed and dissolved in methanol to obtain the standard solution.

### Preparation of the sample solution

Resina Draconis sample powder (0.1 g) was accurately weighed and put into a 15-mL centrifuge tube. After 10 mL of methanol was added, the mixture was extracted for 30 min by ultrasound (240 W) and centrifuged for 5 min. The operation was repeated once, and the residue was washed with 4 mL of methanol and then centrifuged for 5 min. The total extracts were combined in a 25-mL volumetric flask, which was then filled up to the calibration mark with methanol. The extracts were then filtered through a microfiltration membrane (0.20 μm) to obtain the sample solution.

### Validation of the UPLC method

A Resina Draconis sample (sample 12) was used in the validation test. The precision was determined by injecting the same sample solution for six times in 1 day. The repeatability was determined by analyzing six independently sample solution extracted from Resina Draconis of the same batch. The stability test was evaluated by injecting the same sample solution at 0, 2, 4, 8, 12 and 24 h after preparation. The 12 batches of Resina Draconis samples from different regions were analyzed, and the chromatograms were recorded.

### Data analysis

The data analysis was processed by the professional software Similarity Evaluation System for Chromatographic Fingerprint of Traditional Chinese Medicine (Version 2004A), which was recommended by the State Food and Drug Administration (SFDA) of China. This software was used to calculate the correlation coefficients of the chromatographic profiles of 12 batches of Resina Draconis samples, and to generate the simulative mean chromatogram (SMC). The similarities of different chromatographic fingerprints were compared with the SMC.

## Results and discussion

### Optimization of the preparation methods for the sample solution

This experiment compared the preparation methods of sample solution. By comparing the chromatograms obtained from various extraction solvents, it was found that the chromatographic peak, peak area and base line were relatively steady when methanol was used as extraction solvent. By comparing the ultrasound and reflux extraction, no obvious difference in the efficiency was observed between the two extraction methods, so the ultrasound extraction was adopted. Extraction times and cycles were further optimized, and the results demonstrated that exhausted extraction could be achieved when Resina Draconis sample powder of 0.1 g was extracted with 10 mL of methanol by means of sonication for 0.5 h, twice.

### Optimization of the mobile phase

Different mobile phase compositions such as methanol–phosphoric acid aqueous solution, acetonitrile–phosphoric acid aqueous solution, methanol–water and acetonitrile–water system were compared, and acetonitrile–water system was found to give better separation for the chromatographic peaks at a lower column pressure.

### Optimization of the detection wavelength

Full-wavelength scanning from 190 to 400 nm was conducted by the PDA detector, and the results showed that the chromatogram at detection wavelength of 280 nm was abundant in peak information with more obvious characteristics. The five reference components, namely resveratrol, 7,4′-dihydroxyflavone, loureirin A, loureirin B and pterostilbene, were well presented at 280 nm and the baseline was steady. Thus, the detection wavelength was determined to be 280 nm eventually.

### Optimization of the column temperature

The effect of the column temperature (25, 30, 40 and 45 °C) on the chromatographic peak separation was investigated, and it was found that the resolution of the peaks got better at 40 °C UPLC, and the best resolution appeared at 30 °C by HPLC. Thus, 40 and 30 °C were used by UPLC and HPLC, respectively.

### Identification of the common peaks

The UPLC fingerprints generated by the 12 batches of Resina Draconis samples were analyzed and 10 common peaks were found. Among them, five common peaks were identified by comparing the reference substances, namely resveratrol (peak 1), 7,4′-dihydroxyflavone (peak 2), loureirin A (peak 3), loureirin B (peak 4) and pterostilbene (peak 5).

### Comparison of the HPLC and UPLC fingerprints

**Fig. 2:**
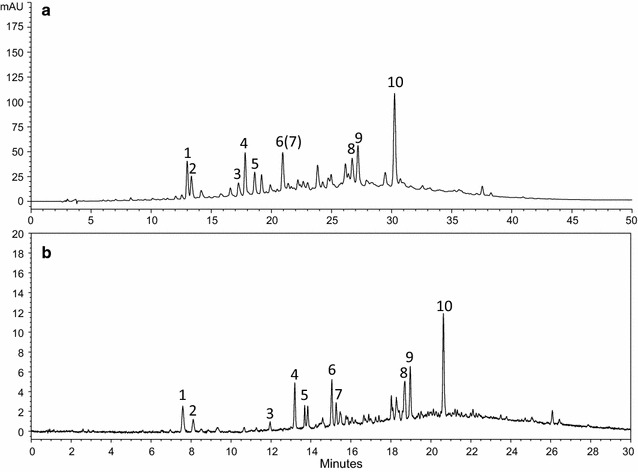
Resina Draconis (sample 12) on conventional HPLC and UPLC at 280 nm: HPLC (**a**); UPLC (**b**). *Peak 1* resveratrol; *Peak 2* 7,4′-dihydroxyflavone; *Peak 3* loureirin A; *Peak 4* loureirin B; *Peak 5* pterostilbene

The chromatograms of the conventional HPLC and UPLC were compared in Fig. 2. For the conventional HPLC, a complete fingerprint chromatogram of Resina Draconis was obtained in 50 min at a flow rate of 1.0 mL/min; but with UPLC, the analysis time was shortened to 30 min at a flow rate of 0.3 mL/min. The analysis efficiency of UPLC is higher, which can remarkably shorten the analysis time and reduce the consumption of mobile phase. Compared with HPLC, the elution requirement of UPLC is simpler, the drift time of chromatographic peak is shorter and the peak of the chromatogram is easier to match. UPLC adopts 1.8 μm superfine chromatographic column filling while HPLC adopts 5 μm chromatographic column filling, so the column efficiency of UPLC is significantly higher than that of HPLC, enabling the separation to be done within 30 min. Compared with the reported UPLC method with separation time of 45 min in the literature [26], the present UPLC method saved the separation time more than 30%. Owing to the high column efficiency of UPLC, the column length of UPLC is relatively shorter than that of HPLC, which is one reason why UPLC has faster separation speed than HPLC. In addition, although fewer injection volumes were used for UPLC analysis, more and stronger peak signals were obtained. These results indicated that UPLC had superior sensitivity and resolution to the conventional HPLC.

### Validation of the UPLC fingerprint method

#### Precision test

For the precision study, the retention time and peak area of the peak 4 (loureirin B) was chosen as the reference, and the relative retention time (RRT) and relative peak area (RPA) of the ten common peaks of all the samples were calculated. The relative standard deviation (RSD) of the RRT of each common peak was found to be less than 0.05%, and the RSD of the RPA of each common peak was less than 4.68% (Table 1), which showed the precision of the UPLC fingerprint method was good.

**Table 1:** The precision, repeatability and stability of the common peaks in Resina Draconis

Peak no.	Precision (RSD, %)		Repeatability (RSD, %)		Stability (RSD, %)	
	RRT	RPA	RRT	RPA	RRT	RPA
1	0.04	4.68	0.14	3.21	0.18	0.76
2	0.05	0.54	0.12	3.7	0.12	2.03
3	0.04	0.9	0.04	1.93	0.08	0.91
4(S)	–	–	–	–	–	–
5	0.02	1.46	0.02	4.5	0.01	0.63
6	0.01	2.2	0.03	3.21	0.02	4.41
7	0.03	2.89	0.01	2.78	0.01	0.52
8	0.02	2.9	0.04	4.79	0.04	0.5
9	0.02	3.95	0.04	2.63	0.04	1.62
10	0.02	0.53	0.04	4.33	0.04	1.01

*RRT* relative retention time, *RPA* relative peak area

#### Repeatability test

The RRT and RPA of the ten common peaks were calculated in the repeatability test. The RSD of the RRT for each peak was less than 0.14%, and the RSD of RPA was less than 4.79%. The two RSD prompted that the repeatability of the UPLC method was satisfied.

#### Stability test

For the stability test, the sample solution has been measured at 0, 2, 4, 6, 8, 12 and 24 h after preparation, and then the RRT and RPA were calculated. The RSD of the RRT was found to be less than 0.18% and the RSD of RPA was less than 4.41%. The results showed that Resina Draconis sample solution was stabile within 24 h.

#### Similarity analysis

Using the Similarity Evaluation System for Chromatographic Fingerprint of Traditional Chinese Medicine (Version 2004A), the RRT and RPA of ten common peaks of 12 batches of Resina Draconis samples were calculated, and the results were listed in Table 2, respectively. The RSD of the RRT was found to be less than 0.52%, while the RSD of the RPA were relatively larger. These results indicated that the retention time of the common peaks were consistent among batches, but the contents of the components among batches significantly varied due to the different origin.

**Table 2:** RRT and RPA of common peaks in the 12 batches of Resina Draconis

Peak no.	1		2		3		4		5		6		7		8		9		10		11		12		RRT	RPA	RRT	RPA
	RRT	RPA	RRT	RPA	RRT	RPA	RRT	RPA	RRT	RPA	RRT	RPA	RRT	RPA	RRT	RPA	RRT	RPA	RRT	RPA	RRT	RPA	RRT	RPA	Mean	Mean	RSD (%)	RSD (%)
1	0.58	0.94	0.58	0.74	0.58	0.85	0.58	0.32	0.58	1.21	0.58	1.03	0.58	2.00	0.58	1.31	0.58	0.68	0.58	0.91	0.58	1.13	0.57	0.93	0.58	1.00	0.52	40.61
2	0.62	0.34	0.62	0.30	0.62	0.46	0.62	0.18	0.62	0.42	0.62	0.67	0.62	1.01	0.62	0.69	0.62	0.27	0.62	0.35	0.62	0.65	0.61	0.45	0.62	0.48	0.29	48.71
3	0.91	0.22	0.91	0.22	0.91	0.75	0.91	0.3	0.91	0.34	0.91	0.41	0.91	2.03	0.91	0.52	0.91	0.22	0.91	0.23	0.91	0.46	0.91	0.21	0.91	0.49	0.10	103.56
4(S)	1	1	1	1	1	1	1	1	1	1	1	1	1	1	1	1	1	1	1	1	1	1	1	1	1	1	0	0
5	1.05	0.39	1.05	0.35	1.05	0.69	1.05	0.21	1.05	0.45	1.05	0.68	1.05	0.93	1.05	0.56	1.05	0.49	1.05	0.42	1.05	0.49	1.05	0.41	1.05	0.51	0.05	37.51
6	1.14	1.10	1.14	0.91	1.14	0.71	1.14	0.23	1.14	1.28	1.14	1.42	1.14	1.37	1.14	1.11	1.14	0.73	1.14	0.91	1.14	1.26	1.14	1.17	1.14	1.02	0.06	33.50
7	1.16	0.47	1.16	0.50	1.16	0.60	1.15	0.35	1.16	0.63	1.16	0.56	1.16	0.93	1.16	0.55	1.16	0.53	1.16	0.49	1.16	0.55	1.16	0.44	1.16	0.55	0.06	25.57
8	1.41	1.25	1.41	1.39	1.41	2.88	1.41	2.36	1.41	1.8	1.41	1.88	1.41	5.03	1.41	1.69	1.41	1.83	1.41	1.33	1.42	1.79	1.42	1.43	1.41	2.06	0.10	50.75
9	1.43	1.24	1.43	1.00	1.43	1.19	1.43	0.37	1.43	1.06	1.43	1.49	1.43	1.76	1.44	1.81	1.44	0.85	1.44	1.03	1.44	1.83	1.44	1.49	1.43	1.26	0.11	34.80
10	1.56	2.41	1.56	2.02	1.56	1.59	1.56	0.35	1.56	2.69	1.56	3.25	1.56	2.01	1.56	3.22	1.56	1.33	1.56	1.92	1.56	3.43	1.56	2.83	1.56	2.25	0.11	40.18

*RRT* relative retention time, *RPA* relative peak area

**Fig. 3:**
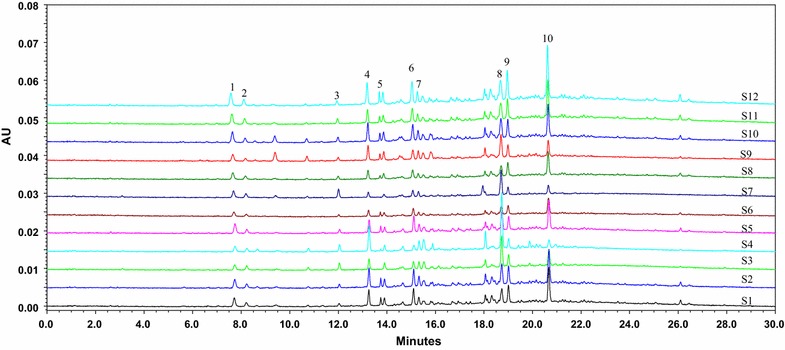
UPLC fingerprints of 12 batches of Resina Draconis at 280 nm.* S1*–*S12* represents Resina Draconis samples numbered from 1 to 12

The overlapped chromatographic fingerprints from 12 batches of Resina Draconis samples were shown in Fig. 3. The results of the similarity analysis were listed in Table 3. Comparison with the SMC, the similarities of the chromatograms of the 12 samples were 0.976, 0.993, 0.955, 0.789, 0.989, 0.995, 0.794, 0.994, 0.847, 0.987, 0.997, 0.986, respectively, which indicated that Resina Draconis samples from different regions were certainly regionally different, but within a moderate and acceptable range. The similarities of the twelve samples showed more similar pattern except for the samples no. 4, 7 and 9, when The threshold was set to 0.9. This difference in similarity may be due to the difference in the sample origin. The samples 4, 7 and 9 were collected from Guangxi province of China, and the remaining nine batches of samples (the samples 1, 2, 3, 5, 6, 8, 10, 11 and 12) were collected from Yunnan province, China (Additional file 1: Table S1).

**Table 3:** Similarities of the 12 batches of Resina Draconis

Sample no.	Similarity
1	0.976
2	0.993
3	0.955
4	0.789
5	0.989
6	0.995
7	0.794
8	0.994
9	0.847
10	0.987
11	0.997
12	0.986

The results of similarity analysis showed that the chemical types of Resina Draconis samples from different regions were basically same, however, the relative contents of the each component were various in some of the samples. This finding demonstrated that the present UPLC fingerprint method could not only distinguish the origin, but also evaluate the relative quality of the Resina Draconis product, which were suitable for the quality control of Resina Draconis.

## Conclusion

A UPLC method for the fingerprinting of Resina Draconis has been established and validated in this study. Compared to the conventional HPLC, the present UPLC method provided a shorter analysis time and higher resolution with good precision, reproducibility and stability. The satisfactory performance of the method was demonstrated through analyzing 12 batches of Resina Draconis samples collected from different regions. To conclude, the UPLC fingerprint method established in the present study was proved to be feasible and reliable, which is extremely helpful in providing a valuable reference for quality control of Resina Draconis and other traditional Chinese medicine.

## Additional file

**Additional file 1: Table S1.** The source of the tested samples.

## Abbreviations

TCM: Traditional Chinese medicine
UPLC: ultra-performance liquid chromatography
HPLC: high-performance liquid chromatography
RRT: relative retention time
RPA: relative peak area
RSD: relative standard deviation
TLC: thin-layer chromatography
HPLC: high-performance liquid chromatography
GC: gas chromatograph
CE: capillary electrophoresis
PDA: photodiode array
DAD: diode array detector
UV: ultraviolet
SFDA: State Food and Drug Administration
SMC: simulative mean chromatogram
