# Supplementary material for: Fingerprint analysis of Resina Draconis by ultra-performance liquid chromatography
Source: Chem Cent J. 2017 Jul 24;11:67. doi: 10.1186/s13065-017-0299-8 (PMC5524661; doi:10.1186/s13065-017-0299-8)
Supplement: Supplementary file 1 — Additional file 1: Table S1. The source of the tested samples. [file 13065_2017_299_MOESM1_ESM.docx]

**Additional file 1: Table S1** The source of the tested samples

| **Sample No.** | **Collection time** | **Place of origin** |
| --- | --- | --- |
| 1 | 2013 | Yunnan Menglian |
| 2 | 2012 | Yunnan Menglian |
| 3 | 2013 | Yunnan Pu'er |
| 4 | 2012 | Guangxi Chongzuo |
| 5 | 2014 | Yunnan Zhenkang |
| 6 | 2015 | Yunnan Pu'er |
| 7 | 2014 | Guangxi Yaotou Wei |
| 8 | 2013 | Yunnan Zhenkang |
| 9 | 2014 | Guangxi Baise |
| 10 | 2014 | Yunnan Pu'er |
| 11 | 2015 | Yunnan Menglian |
| 12 | 2013 | Yunnan Zhenkang |
